# A Review of Artificial Intelligence and Robotics in Transformed Health Ecosystems

**DOI:** 10.3389/fmed.2022.795957

**Published:** 2022-07-06

**Authors:** Kerstin Denecke, Claude R. Baudoin

**Affiliations:** ^1^Institute for Medical Information, Bern University of Applied Sciences, Bern, Switzerland; ^2^Object Management Group, Needham, MA, United States

**Keywords:** artificial intelligence, robotics, healthcare, personalized medicine, P5 medicine

## Abstract

Health care is shifting toward become proactive according to the concept of P5 medicine–a predictive, personalized, preventive, participatory and precision discipline. This patient-centered care heavily leverages the latest technologies of artificial intelligence (AI) and robotics that support diagnosis, decision making and treatment. In this paper, we present the role of AI and robotic systems in this evolution, including example use cases. We categorize systems along multiple dimensions such as the type of system, the degree of autonomy, the care setting where the systems are applied, and the application area. These technologies have already achieved notable results in the prediction of sepsis or cardiovascular risk, the monitoring of vital parameters in intensive care units, or in the form of home care robots. Still, while much research is conducted around AI and robotics in health care, adoption in real world care settings is still limited. To remove adoption barriers, we need to address issues such as safety, security, privacy and ethical principles; detect and eliminate bias that could result in harmful or unfair clinical decisions; and build trust in and societal acceptance of AI.

## The Need for AI and Robotics in Transformed Health Ecosystems

“Artificial intelligence (AI) is the term used to describe the use of computers and technology to simulate intelligent behavior and critical thinking comparable to a human being” (1). Machine learning enables AI applications to automatically (i.e., without being explicitly programmed for) improving their algorithms through experiences gained by cognitive inputs or by the use of data. AI solutions provide data and knowledge to be used by humans or other technologies. The possibility of machines behaving in such a way was originally raised by Alan Turing and further explored starting in the 1950s. Medical expert systems such as MYCIN, designed in the 1970s for medical consultations (2), were internationally recognized a revolution supporting the development of AI in medicine. However, the clinical acceptance was not very high. Similar disappointments across multiple domains led to the so-called “AI winter,” in part because rule-based systems do not allow the discovery of unknown relationships and in part because of the limitations in computing power at the time. Since then, computational power has increased enormously.

Over the centuries, we have improved our knowledge about structure and function of the human body, starting with the organs, tissues, cells sub-cell components etc. Meanwhile, we could advance it up to the molecular and sub-molecular level, including protein coding genes, DNA sequences, non-coding RNA etc. and their effects and behavior in the human body. This has resulted in a continuously improving understanding of the biology of diseases and disease progressions (3). Nowadays, biomedical research and clinical practice are struggling with the size and complexity of the data produced by sequencing technologies, and how to derive from it new diagnoses and treatments. Experiment results, often hidden in clinical data warehouses, must be aggregated, analyzed, and exploited to derive our new, detailed and data-driven knowledge of diseases and enable better decision making.

New tools based on AI have been developed to predict disease recurrence and progression (4) or response to treatment; and robotics, often categorized as a branch of AI, plays an increasing role in patient care. In a medical context, AI means for example imitating the decision-making processes of health professionals (1). In contrast to AI that generates data, robotics provides touchable outcomes or realize physical tasks. AI and robotics use knowledge and patient data for various tasks such as: diagnosis; planning of surgeries; monitoring of patient physical and mental wellness; basic physical interventions to improve patient independence during physical or mental deterioration. We will review concrete realizations in a later section of this paper.

These advances are causing a revolution in health care, enabling it to become proactive as called upon by the concept of P5 medicine –a predictive, personalized, preventive, participatory and precision discipline (5). AI can help interpret personal health information together with other data to stratify the diseases to predict, stop or treat their progression.

In this paper, we describe the impact of AI and robotics on P5 medicine and introduce example use cases. We then discuss challenges faced by these developments. We conclude with recommendations to help AI and robotics transform health ecosystems. We extensively refer to appropriate literature for details on the underlying methods and technologies. Note that we concentrate on applications in the care setting and will not address in more detail the systems used for the education of professionals, logistics, or related to facility management–even though there are clearly important applications of AI in these areas.

## Classification of AI and Robotic Systems in Medicine

**Figure 1:**
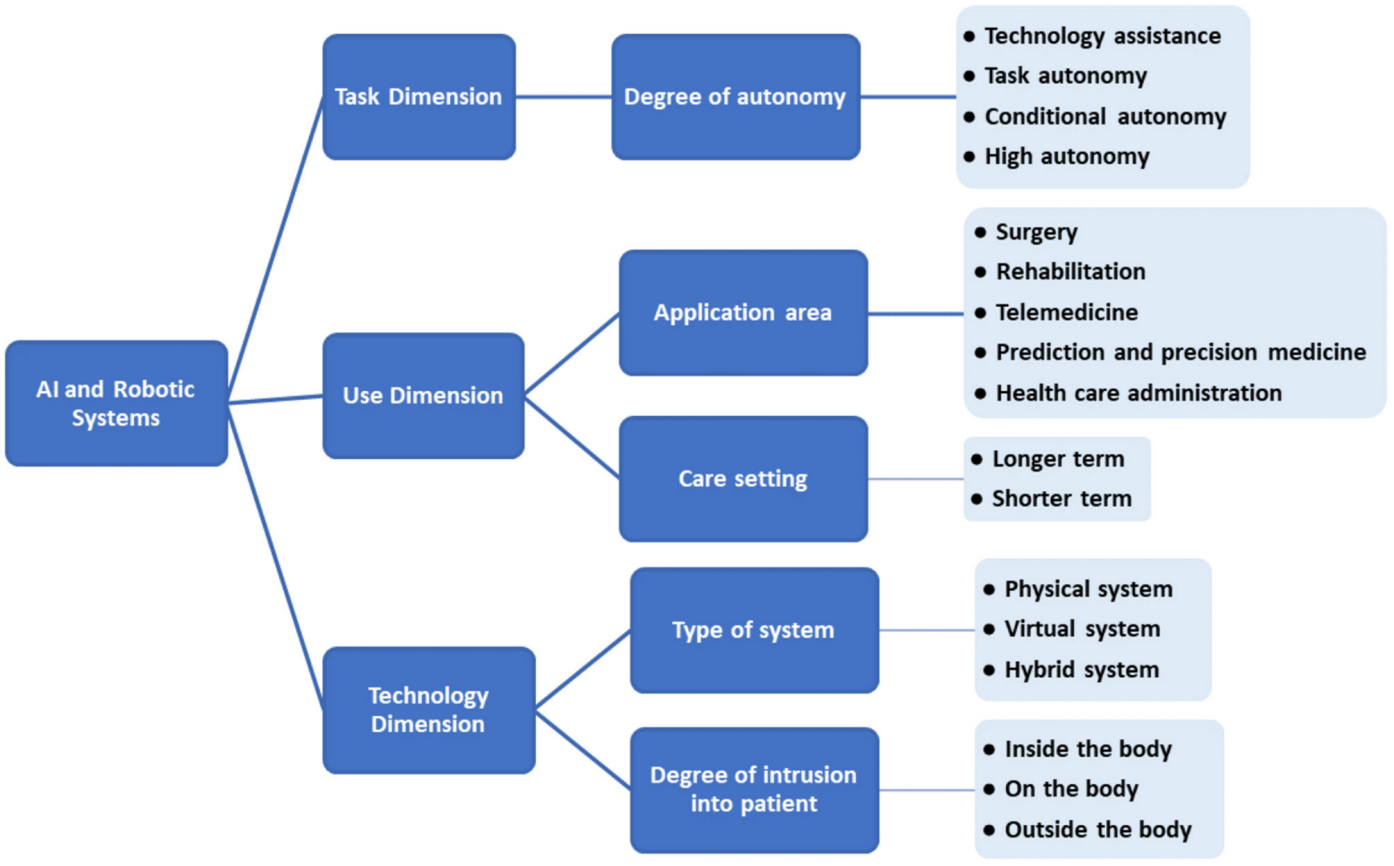
Categorization of systems based on AI and robotics in health care.

We can classify the landscape of AI and robotic systems in health care according to different dimensions (Figure 1): use, task, technology. Within the “use” dimension, we can further distinguish the application area or the care setting. The “task” dimension is characterized by the system's degree of autonomy. Finally, regarding the “technology” dimension, we consider the degree of intrusion into a patient and the type of system. Clearly, this is a simplification and aggregation: AI algorithms as such will not be located in a patient etc.

### Classification Based on Type of System

We can distinguish two types of such systems: virtual and physical (6).

- Virtual systems (relating to AI systems) range from applications such as electronic health record (EHR) systems, or text and data mining applications, to systems supporting treatment decisions.
- Physical systems relate to robotics and include robots that assist in performing surgeries, smart prostheses for handicapped people, and physical aids for elderly care.

There can also be hybrid systems combining AI with robotics, such as social robots that interact with users or microrobots that deliver drugs inside the body.

**Figure 2:**
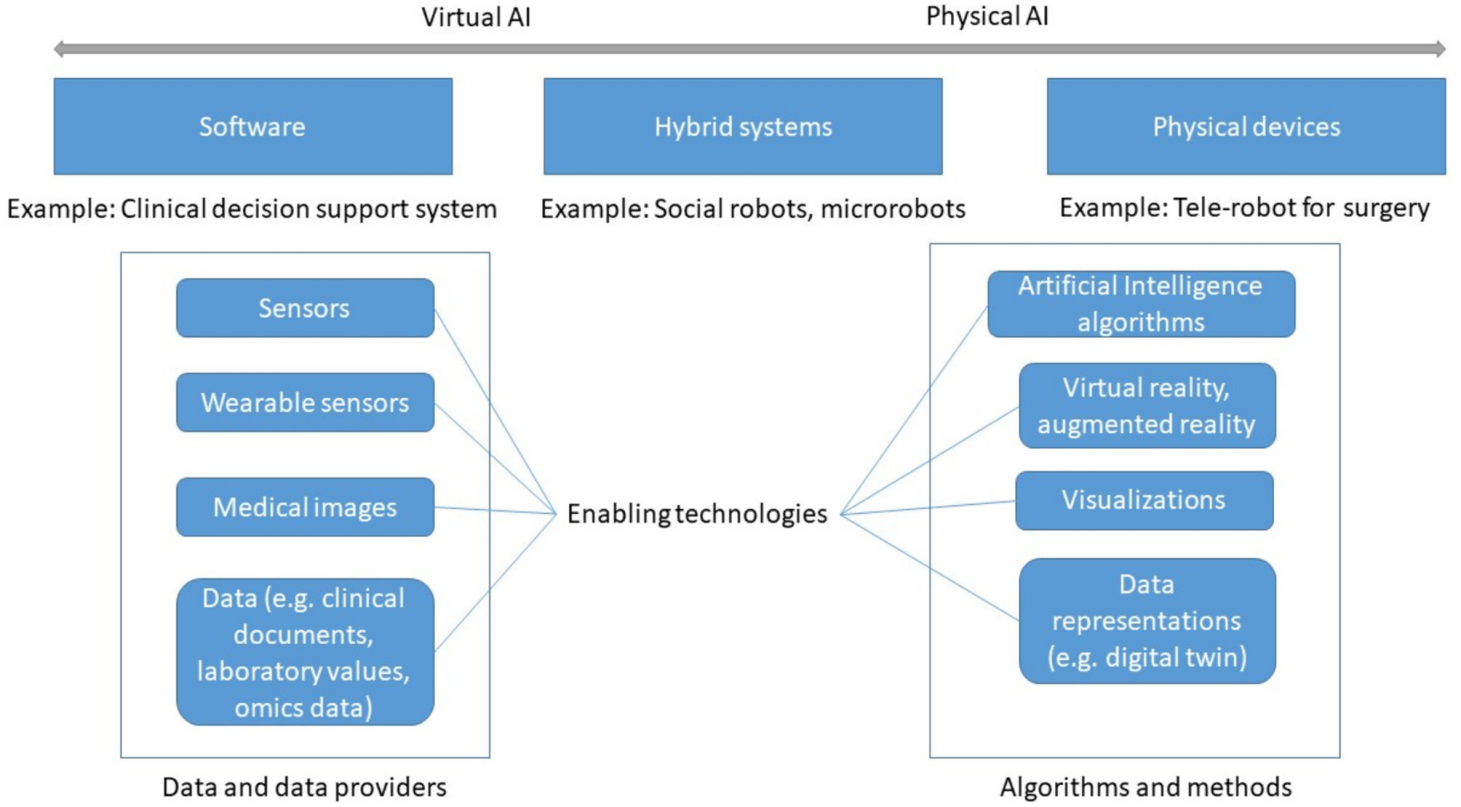
Types of AI-based systems and enabling technologies.

All these systems exploit enabling technologies that are *data* and *algorithms* (see Figure 2). For example, a robotic system may collect data from different sensors–visual, physical, auditory or chemical. The robot's processor manipulates, analyzes, and interprets the data. Actuators enable the robot to perform different functions including visual, physical, auditory or chemical responses.

#### Data

Two kinds of data are required: data that captures the knowledge and experience gained by the system during diagnosis and treatment, usually through machine learning; and individual patient data, which AI can assess and analyze to derive recommendations. Data can be obtained from physical sensors (wearable, non-wearable), from biosensors (7), or from other information systems such as an EHR application. From the collected data, digital biomarkers can be derived that AI can analyze and interpret (8).

#### Algorithms

AI-specific algorithms and methods allow data analysis, reasoning, and prediction. AI consists of a growing number of subfields such as machine learning (supervised, unsupervised, and reinforcement learning), machine vision, natural language processing (NLP) and more. NLP enables computers to process and understand natural language (written or spoken). Machine vision or computer vision extracts information from images. An authoritative taxonomy of AI does not exist yet, although several standards bodies have started addressing this task.

AI methodologies can be divided into knowledge-based AI and data-driven AI (9).

- *Knowledge-based AI* models human knowledge by asking experts for relevant concepts and knowledge they use to solve problems. This knowledge is then formalized in software (9). This is the form of AI closest to the original expert systems of the 1970s.
- *Data-driven AI* starts from large amounts of data, which are typically processed by machine learning methods to learn patterns that can be used for prediction. Virtual or augmented reality and other types of visualizations can be used to present and explore data, which helps understand relations among data items that are relevant for diagnosis (10).

To more fully exploit the knowledge captured in computerized models, the concept of *digital twin* has gained traction in the medical field (11). The terms “digital patient model,” “virtual physiological human,” or “digital phenotype” designate the same idea. A digital twin is a virtual model fed by information coming from wearables (12), omics, and patient records. Simulation, AI and robotics can then be applied to the digital twin to learn about the disease progression, to understand drug responses, or to plan surgery, before intervening on the actual patient or organ, effecting a significant digital transformation of the health ecosystems. Virtual organs (e.g., a digital heart) are an application of this concept (13). A digital twin can be customized to an individual patient, thus improving diagnosis.

Regardless of the specific kind of AI, there are some requirements that all AI and robotic systems must meet. They must be:

- *Adaptive*. Transformed health ecosystems evolve rapidly, especially since according to P5 principles they adapt treatment and diagnosis to individual patients.
- *Context-aware*. They must infer the current activity state of the user and the characteristics of the environment in order to manage information content and distribution.
- *Interoperable*. A system must be able to exchange data and knowledge with other ones (14). This requires common semantics between systems, which is the object of standard terminologies, taxonomies or ontologies such as SNOMED CT. NLP can also help with interoperability (15).

### Classification Based on Degree of Autonomy

**Figure 3:**
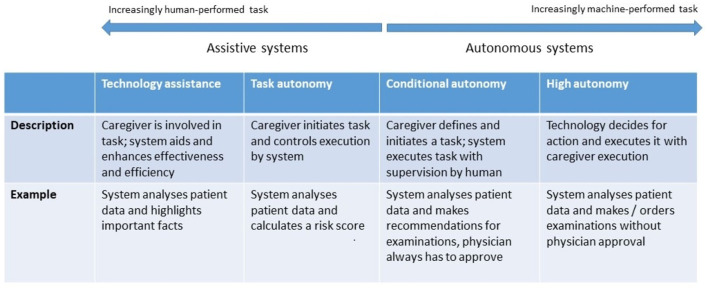
Levels of autonomy of robotic and AI systems. [following models proposed by (16)].

AI and robotic systems can be grouped along an assistive-to-autonomous axis (Figure 3). Assistive systems augment the capabilities of their user by aggregating and analyzing data, performing concrete tasks under human supervision [for example, a semiautonomous ultrasound scanner (17)], or learning how to perform tasks from a health professional's demonstrations. For example, a robot may learn from a physiotherapist how to guide a patient through repetitive rehabilitation exercises (18).

Autonomous systems respond to real world conditions, make decisions, and perform actions with minimal or no interaction with a human (19). They be encountered in a clinical setting (autonomous implanted devices), in support functions to provide assistance^1^ (carrying things around in a facility), or to automate non-physical work, such as a digital receptionist handling patient check-in (20).

### Classification Based on Application Area

The diversity of users of AI and robotics in health care implies an equally broad range of application areas described below.

#### Robotics and AI for Surgery

Robotics-assisted surgery, “the use of a mechanical device to assist surgery in place of a human-being or in a human-like way” (21) is rapidly impacting many common general surgical procedures, especially minimally invasive surgery. Three types of robotic systems are used in surgery:

- Active systems undertake pre-programmed tasks while remaining under the control of the operating surgeon;
- Semi-active systems allow a surgeon to complement the system's pre-programmed component;
- Master–slave systems lack any autonomous elements; they entirely depend on a surgeon's activity. In laparoscopic surgery or in teleoperation, the surgeon's hand movements are transmitted to surgical instruments, which reproduce them.

Surgeons can also be supported by navigation systems, which localize positions in space and help answer a surgeon's anatomical orientation questions. Real-time tracking of markers, realized in modern surgical navigation systems using a stereoscopic camera emitting infrared light, can determine the 3D position of prominent structures (22).

#### Robotics and AI for Rehabilitation

Various AI and robotic systems support rehabilitation tasks such as monitoring, risk prevention, or treatment (23). For example, fall detection systems (24) use smart sensors placed within an environment or in a wearable device, and automatically alert medical staff, emergency services, or family members if assistance is required. AI allows these systems to learn the normal behavioral patterns and characteristics of individuals over time. Moreover, systems can assess environmental risks, such as household lights that are off or proximity to fall hazards (e.g., stairwells). Physical systems can provide physical assistance (e.g., lifting items, opening doors), monitoring, and therapeutic social functions (25). Robotic rehabilitation applications can provide both physical and cognitive support to individuals by monitoring physiological progress and promoting social interaction. Robots can support patients in recovering motions after a stroke using exoskeletons (26), or recovering or supplementing lost function (27). Beyond directly supporting patients, robots can also assist caregivers. An overview on home-based rehabilitation robots is given by Akbari et al. (28). Virtual reality and augmented reality allow patients to become immersed within and interact with a 3D model of a real or imaginary world, allowing them to practice specific tasks (29). This has been used for motor function training, recovery after a stroke (30) and in pain management (31).

#### Robotics and AI for Telemedicine

Systems supporting telemedicine support among others the triage, diagnostic, non-surgical treatment, surgical treatment, consultation, monitoring, or provision of specialty care (32).

- Medical triage assesses current symptoms, signs, and test results to determine the severity of a patient's condition and the treatment priority. An increasing number of mobile health applications based on AI are used for diagnosis or treatment optimization (33).
- Smart mobile and wearable devices can be integrated into “smart homes” using Internet-of-Things (IoT) technologies. They can collect patient and contextual data, assist individuals with everyday functioning, monitor progress toward individualized care and rehabilitation goals, issue reminders, and alert care providers if assistance is required.
- Telemedicine for specialty care includes additional tools to track mood and behavior (e.g., pain diaries), AI-based chatbots can mitigate social isolation in home care environments^2^ by offering companionship and emotional support to users, noting if they are not sleeping well, in pain or depressed, which could indicate a more complex mental condition (34).
- Beyond this, there are physical systems that can deliver specialty care: Robot DE NIRO can interact naturally, reliably, and safely with humans, autonomously navigate through environments on command, intelligently retrieve or move objects (35).

#### Robotics and AI for Prediction and Precision Medicine

Precision medicine considers the individual patients, their genomic variations as well as contributing factors (age, gender, ethnicity, etc.), and tailors interventions accordingly (8). Digital health applications can also incorporate data such as emotional state, activity, food intake, etc. Given the amount and complexity of data this requires, AI can learn from comprehensive datasets to predict risks and identify the optimal treatment strategy (36). Clinical decision support systems (CDSS) that integrate AI can provide differential diagnoses, recognize early warning signs of patient morbidity or mortality, or identify abnormalities in radiological images or laboratory test results (37). They can increase patient safety, for example by reducing medication or prescription errors or adverse events and can increase care consistency and efficiency (38). They can support clinical management by ensuring adherence to the clinical guidelines or automating administrative functions such as clinical and diagnostic encoding (39), patient triage or ordering of procedures (37).

#### AI and Agents for Management and Support Tasks

NLP applications, such as voice transcription, have proved helpful for clinical note-taking (40), compiling electronic health records, automatically generating medical reports from patient-doctor conversations or diagnostic reports (41). AI algorithms can help retrieving context-relevant patient data. Concept-based information retrieval can improve search accuracy and retrieval speed (42). AI algorithms can improve the use and allocation of hospital resources by predicting the length of stay of patients (43) or risk of re-admission (44).

### Classification Based on Degree of Intrusion Into a Patient

Robotic systems can be used inside the body, on the body or outside the body. Those applied *inside* the body include microrobots (45), surgical robots and interventional robots. Microrobots are sub-millimeter untethered devices that can be propelled for example by chemical reactions (46), or physical fields (47). They can move unimpeded through the body and perform tasks such as targeted therapy (localized delivery of drugs) (48).

Microrobots can assist in physical surgery, for example by drilling through a blood clot or by opening up obstructions in the urinary tract to restore normal flow (49). They can provide directed local tissue heating to destroy cancer cells (50). They can be implanted to provide continuous remote monitoring and early awareness of an emerging disease.

Robotic prostheses, orthoses and exoskeletons are examples of robotic systems worn *on* the body. Exoskeletons are wearable robotic systems that are tightly physically coupled with a human body to provide assistance or enhance the wearer's physical capabilities (51). While they have often been developed for applications outside of health care, they can help workers with physically demanding tasks such as moving patients (52) or assist people with muscle weakness or movement disorders. Wearable technology can also be used to measure and transmit data about vital signs or physical activity (19).

Robotic systems applied *outside* the body can help avoid direct contact when treating patients with infectious diseases (53), assist in surgery (as already mentioned), including remote surgical procedures that leverage augmented reality (54) or assist providers when moving patients (55).

### Classification Based on Care Setting

Another dimension of AI and robotics is the duration of their use, which directly correlates with the location of use. Both can significantly influence the requirements, design, and technology components of the solution. In a longer-term care setting, robotics can be used in a patient's home (e.g., for monitoring of vital signs) or for treatment in a nursing home. Shorter-term care settings include inpatient hospitals, palliative care facilities or inpatient psychiatric facilities. Example applications are listed in Table 1.

**Table 1:** Classification by care setting.

**Care setting**		**Description**	**Example**
**Longer term**	Home care	Personal living environment	Remote monitoring of individuals for identifying early indications of heart failure decompensation, which allows for optimization of therapy to prevent hospitalizations (56)
	Assisted living facility	Residential facility with self-contained living units; site support 24 x 7 and capacity to arrange health care services	A smart kitchen for ambient assisted living (57)
	Nursing home	Facility providing residential accommodation with health care	Social robots to treat individuals with dementia in order to improve symptoms (58)
**Shorter term**	Inpatient hospital	Provides diagnostic, therapeutic and rehabilitation services by or under supervision of physicians	Virtual nurse for hospital discharge planning (59)
	Hospice	Facility that offers palliative and supportive care for terminally ill persons and their families	Conversational agent to collect patient reported outcome measures from individuals in palliative care (60)
	Inpatient psychiatric facility	Inpatient psychiatric services for the diagnosis and treatment of mental health disorders	AI to predict risk or severity of depression (61)

*Selected care settings where robotic systems may be used [adapted from (62)]*.

## Sample Realizations

Having seen how to classify AI and robotic systems in health care, we turn to recent concrete achievements that illustrate their practical application and achievements already realized. This list is definitely not exhaustive, but it illustrates the fact that we're no longer purely at the research or experimentation stage: the technology is starting to bear fruit in a very concrete way–that is, by improving outcomes–even when only in the context of clinical trials prior to regulatory approval for general use.

### Sepsis Onset Prediction

Sepsis was recently identified as *the* leading cause of death worldwide, surpassing even cancer or cardiovascular diseases.^3^ And while timely diagnosis and treatment are difficult in other care settings, it is also the leading cause of death in hospitals in the United States (Sepsis Fact Sheet^4^) A key reason is the difficulty of recognizing precursor symptoms early enough to initiate effective treatment. Therefore, early onset prediction promises to save millions of lives each year. Here are four such projects:

- Bayesian Health^5^, a startup founded by a researcher at Johns Hopkins University, applied its model to a test population of hospital patients and correctly identified 82% of the 9,800 patients who later developed sepsis.
- Dascena, a California startup, has been testing its software on large cohorts of patients since 2017, achieving significant improvements in outcomes (63).
- Patchd^6^ uses wearable devices and deep learning to predict sepsis in high-risk patients. Early studies have shown that this technology can predict sepsis 8 h earlier, and more accurately, than under existing standards of care.
- A team of researchers from Singapore developed a system that combines clinical measures (structured data) with physician notes (unstructured data), resulting in improved early detection while reducing false positives (64).

### Monitoring Systems in the Intensive Care Unit

For patients in an ICU, the paradox is that large amounts of data are collected, displayed on monitors, and used to trigger alarms, but these various data streams are rarely used together, nor can doctors or nurses effectively observe all the data from all the patients all the time.

This is an area where much has been written, but most available information points to studies that have not resulted in actual deployments. A survey paper alluded in particular to the challenge of achieving effective collaboration between ICU staff and automated processes (65).

In one application example, machine learning helps resolving the asynchrony between a mechanical ventilator and the patient's own breathing reflexes, which can cause distress and complicate recovery (66).

### Tumor Detection From Image Analysis

This is another area where research has provided evidence of the efficacy of AI, generally not employed alone but rather as an advisor to a medical professional, yet there are few actual deployments at scale.

These applications differ based on the location of the tumors, and therefore on the imaging techniques used to observe them. AI makes the interpretation of the images more reliable, generally by pinpointing to the radiologists areas they might otherwise overlook.

- In a study performed in Korea, AI appeared to improve the recognition of lung cancer in chest X-rays (67). AI by itself performed better than unaided radiologists, and the improvement was greater when AI was used as an aid by radiologists. Note however that the sample size was fairly small.
- Several successive efforts aimed to use AI to classify dermoscopic images to discriminate between benign nevi and melanoma (68).

### AI for COVID-19 Detection

The rapid and tragic emergence of the COVID-19 disease, and its continued evolution at the time of this writing, have mobilized many researchers, including the AI community. This domain is naturally divided into two areas, diagnostic and treatment.

An example of AI applied to COVID-19 diagnostic is based on an early observation that the persistent cough that is one of the common symptoms of the disease “sounds different” from the cough caused by other ailments, such as the common cold. The MIT Opensigma project^7^ has “crowdsourced” sound recordings of coughs from many people, most of whom do not have the disease while some know that they have it or had it. Several similar projects have been conducted elsewhere (69).

Another effort used AI to read computer tomography images to provide a rapid COVID-19 test, reportedly achieving over 90% accuracy in 15 s (70). Curiously, after this news was widely circulated in February-March 2020, nothing else was said for several months. Six months later, a blog post^8^ from the University of Virginia radiology and medical department asserted that “CT scans and X-rays have a limited role in diagnosing coronavirus.” The approach pioneered in China may have been the right solution at a specific point in time (many cases concentrated in a small geographical area, requiring a massive detection effort before other rapid tests were available), thus overriding the drawbacks related to equipment cost and patient exposure to radiation.

### Patient Triage and Symptom Checkers

While the word triage immediately evokes urgent decisions about what interventions to perform on acutely ill patients or accident victims, it can also be applied to remote patient assistance (e.g., telehealth applications), especially in areas underserved by medical staff and facilities.

In an emergency care setting, where triage decisions can result in the survival or death of a person, there is a natural reluctance to entrust such decisions to machines. However, AI as a predictor of outcomes could serve as an assistant to an emergency technician or doctor. A 2017 study of emergency room triage of patients with acute abdominal pain only showed an “acceptable level of accuracy” (71), but more recently, the Mayo Clinic introduced an AI-based “digital triage platform” from Diagnostic Robotics^9^ to “perform clinical intake of patients and suggest diagnoses and hospital risk scores.” These solutions can now be delivered by a website or a smartphone app, and have evolved from decision trees designed by doctors to incorporate AI.

### Cardiovascular Risk Prediction

Google Research announced in 2018 that it has achieved “prediction of cardiovascular risk factors from retinal fundus photographs via deep learning” with a level of accuracy similar to traditional methods such as blood tests for cholesterol levels (72). The novelty consists in the use of a neural network to analyze the retina image, resulting in more power at the expense of explainability.

In practice, the future of such a solution is unclear: certain risk factors could be assessed from the retinal scan, but those were often factors that could be measured directly anyway–such as from blood pressure.

### Gait Analysis

Many physiological and neurological factors affect how someone walks, given the complex interactions between the sense of touch, the brain, the nervous system, and the muscles involved. Certain conditions, in particular Parkinson's disease, have been shown to affect a person's gait, causing visible symptoms that can help diagnose the disease or measure its progress. Even if an abnormal gait results from another cause, an accurate analysis can help assess the risk of falls in elderly patients.

Compared to other applications in this section, gait analysis has been practiced for a longer time (over a century) and has progressed incrementally as new motion capture methods (film, video, infrared cameras) were developed. In terms of knowledge representation, see for example the work done at MIT twenty years ago (73). Computer vision, combined with AI, can considerably improve gait analysis compared to a physician's simple observation. Companies such as Exer^10^ offer solutions that physical therapists can use to assess patients, or that can help monitor and improve a home exercise program. This is an area where technology has already been deployed at scale: there are more than 60 clinical and research gate labs^11^ in the U.S. alone.

### Home Care Robots

Robots that provide assistance to elderly or sick persons have been the focus of research and development for several decades, particularly in Japan due to the country's large aging population with above-average longevity. “Elder care robots” can be deployed at home (with cost being an obvious issue for many customers) or in senior care environments (74), where they will help alleviate a severe shortage of nurses and specialized workers, which cannot be easily addressed through the hiring of foreign help given the language barrier.

The types of robots used in such settings are proliferating. They range from robots that help patients move or exercise, to robots that help with common tasks such as opening the front door to a visitor or bringing a cup of tea, to robots that provide psychological comfort and even some form of conversation. PARO, for instance, is a robotic bay seal developed to provide treatment to patients with dementia (75).

### Biomechatronics

Biomechatronics combines biology, mechanical engineering, and electronics to design assistive devices that interpret inputs from sensors and send commands to actuators–with both sensors and actuators attached in some manner to the body. The sensors, actuators, control system, and the human subject form together a closed-loop control system.

Biomechatronic applications live at the boundary of prosthetics and robotics, for example to help amputees achieve close-to-normal motion of a prosthetic limb. This work has been demonstrated for many years, with impressive results, at the MIT Media Lab under Prof. Hugh Herr^12^ However, those applications have rarely left the lab environment due to the device cost. That cost could be lowered by production in large quantities, but coverage by health insurance companies or agencies is likely to remain problematic.

### Mapping of Use Cases to Classification

Table 2 shows a mapping of the above use cases to the classification introduced in the first section of this paper.

**Table 2:** Mapping of use cases to our classification.

**Use case**	**Application area**	**Autonomy**	**Intrusion**	**Care setting**
Sepsis onset prediction	Prediction and precision medicine	Technology assistance	Outside the body	Shorter term
Monitoring in the ICU	Surgery	Technology assistance	Outside the body	Shorter term
Tumor detection from image analysis	Prediction and precision medicine	Technology assistance	Outside the body	Shorter term
COVID-19 detection	Prediction and precision medicine	Technology assistance	Outside the body	Shorter term
Patient triage and symptom checker	Prediction and precision medicine	Task autonomy	Outside the body	Shorter term
Cardiovascular risk prediction	Prediction and precision medicine	Technology assistance	Outside the body	Shorter term
Gait analysis	Prediction and precision medicine, Rehabilitation	Technology assistance	Outside the body	Shorter term
Home care robots	Telemedicine	Technology assistance, task autonomy	Outside the body	Longer term
Biomechatronics	Rehabilitation	Task autonomy	On the body	Longer term

## Adoption Challenges to AI and Robotics in Health Care

While the range of opportunities, and the achievements to date, of robotics and AI are impressive as seen above, multiple issues impede their deployment and acceptance in daily practice.

Issues related to trust, security, privacy and ethics are prevalent across all aspects of health care, and many are discussed elsewhere in this issue. We will therefore only briefly mention those challenges that are unique to AI and robotics.

### Resistance to Technology

Health care professionals may ignore or resist new technologies for multiple reasons, including actual or perceived threats to professional status and autonomy (76), privacy concerns (77) or the unresolved legal and ethical questions of responsibility (78). The issues of worker displacement by robots are just as acute in health care as in other domains. Today, while surgery robots operate increasingly autonomously, humans still perform many tasks and play an essential role in determining the robot's course of operation (e.g., for selecting the process parameters or for the positioning of the patient) (79). This allocation of responsibilities is bound to evolve.

### Transparency and Explainability

Explainability is “a characteristic of an AI-driven system allowing a person to reconstruct why a certain AI came up with the presented prediction” (80). In contrast to rule-based systems, AI-based predictions can often not be explained in a human-intelligible manner, which can hide errors or bias (the “black box problem” of machine learning). The explainability of AI models is an ongoing research area. When information on the reasons for an AI-based decision is missing, physicians cannot judge the reliability of the advice and there is a risk to patient safety.

### Responsibility, Accountability and Liability

Who is responsible when the AI or robot makes mistakes or creates harm in patients? Is it the programmer, manufacturer, end user, the AI/robotic system itself, the provider of the training dataset, or something (or someone) else? The answer depends on the system's degree of autonomy. The European Parliament's 2017 Resolution on AI (81) assigns legal responsibility for an action of an AI or robotic system to a human actor, which may be its owner, developer, manufacturer or operator.

### Data Protection

Machine learning requires access to large quantities of data regarding patients as well as healthy people. This raises issues regarding the ownership of data, protection against theft, compliance with regulations such as HIPAA in the U.S. (82) or GDPR for European citizens (83), and what level of anonymization of data is necessary and possible. Regarding the last point, AI models could have unintended consequences, and the evolution of science itself could make patient re-identification possible in the future.

### Data Quality and Integration

Currently, the reliability and quality of data received from sensors and digital health devices remain uncertain (84)–a fact that future research and development must address. Datasets in medicine are naturally imperfect (due to noise, errors in documentation, incompleteness, differences in documentation granularities, etc.), hence it is impossible to develop error-free machine learning models (80). Furthermore, without a way to quickly and reliably integrate the various data sources for analysis, there is lost potential for fast diagnosis by AI algorithms.

### Safety and Security

Introducing AI and robotics into the delivery of health care is likely to create new risks and safety issues. Those will exist even under normal functioning circumstances, when they may be due to design, programming or configuration errors, or improper data preparation (85).

These issues only get worse when considering the probability of cyberattacks:

- Patient data may be exposed or stolen, perhaps by scammers who want to exploit it for profit.
- Security vulnerabilities in robots that interact directly with patients may cause malfunctions that physically threaten the patient or professional. The robot may cause harm directly, or indirectly by giving a surgeon incorrect feedback. In case of unexpected robot behavior, it may be unclear to the user whether the robot is functioning properly or is under attack (86).

The EU Commission recently drafted a legal framework^13^ addressing the risks of AI (not only in health care) in order to improve the safety of and trust in AI. The framework distinguishes four levels of risks: unacceptable risk, high risk, limited risk and minimal risk. AI systems with unacceptable risks will be prohibited, high-risk ones will have to meet strict obligations before release (e.g., risk assessment and mitigation, traceability of results). Limited-risk applications such as chatbots (which can be used in telemedicine) will require “labeling” so that users are made aware that they are interacting with an AI-powered system.

### Biases

While P5 medicine aims at considering multiple factors–ethnicity, gender, socio-economic background, education, etc.–to come up with individualized care, current implementations of AI often demonstrate potential biases toward certain patient groups of the population. The training datasets may have under-represented those groups, or important features may be distributed differently across groups–for example, cardiovascular disease or Parkinson's disease progress differently in men and women (87), so the corresponding features will vary. These causes result in undesirable bias and “unintended of unnecessary discrimination” of subgroups (88).

On the flip side, careful implementations of AI could explicitly consider gender, ethnicity, etc. differences to achieve more effective treatments for patients belonging to those groups. This can be considered “desirable bias” that counteracts the undesirable kind (89) and gets us closer to the goals of P5 medicine.

### Trust–An Evolving Relationship

**Figure 4:**
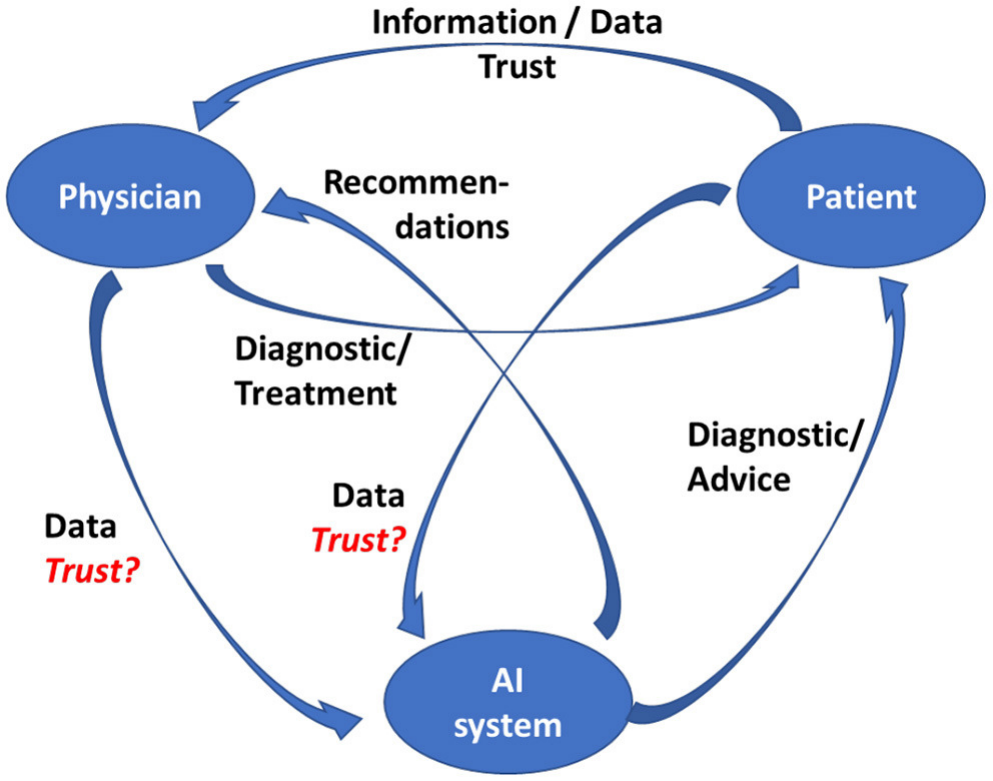
Physician-patient-AI relationship.

The relationship between patients and medical professionals has evolved over time, and AI is likely to impact it by inserting itself into the picture (see Figure 4). Although AI and robotics are performing well, human surveillance is still essential. Robots and AI algorithms operate logically, but health care often requires acting empathically. If doctors become intelligent users of AI, they may retain the trust associated with their role, but most patients, who have a limited understanding of the technologies involved, would have much difficulty in trusting AI (90). Conversely, reliable and accurate diagnosis and beneficial treatment, and appropriate use of AI and robotics by the physician can strengthen the patient's trust (91).

This assumes of course that the designers of those systems adhere to established guidelines for trustworthy AI in the first place, which includes such requirements as creating systems that are lawful, ethical, and robust (92, 93).

## AI and Robotics for Transformed Health Care–A Converging Path

We can summarize the previous sections as follows:

1. There are many types of AI applications and robotic systems, which can be introduced in many aspects of health care.
2. AI's ability to digest and process enormous amounts of data, and derive conclusions that are not obvious to a human, holds the promise of more personalized and predictive care–key goals of P5 medicine.
3. There have been, over the last few years, a number of proof-of-concept and pilot projects that have exhibited promising results for diagnosis, treatment, and health maintenance. They have not yet been deployed at scale–in part because of the time it takes to fully evaluate their efficacy and safety.
4. There is a rather daunting list of challenges to address, most of which are not purely technical–the key one being demonstrating that the systems are effective and safe enough to warrant the confidence of both the practitioners and their patients.

**Figure 5:**
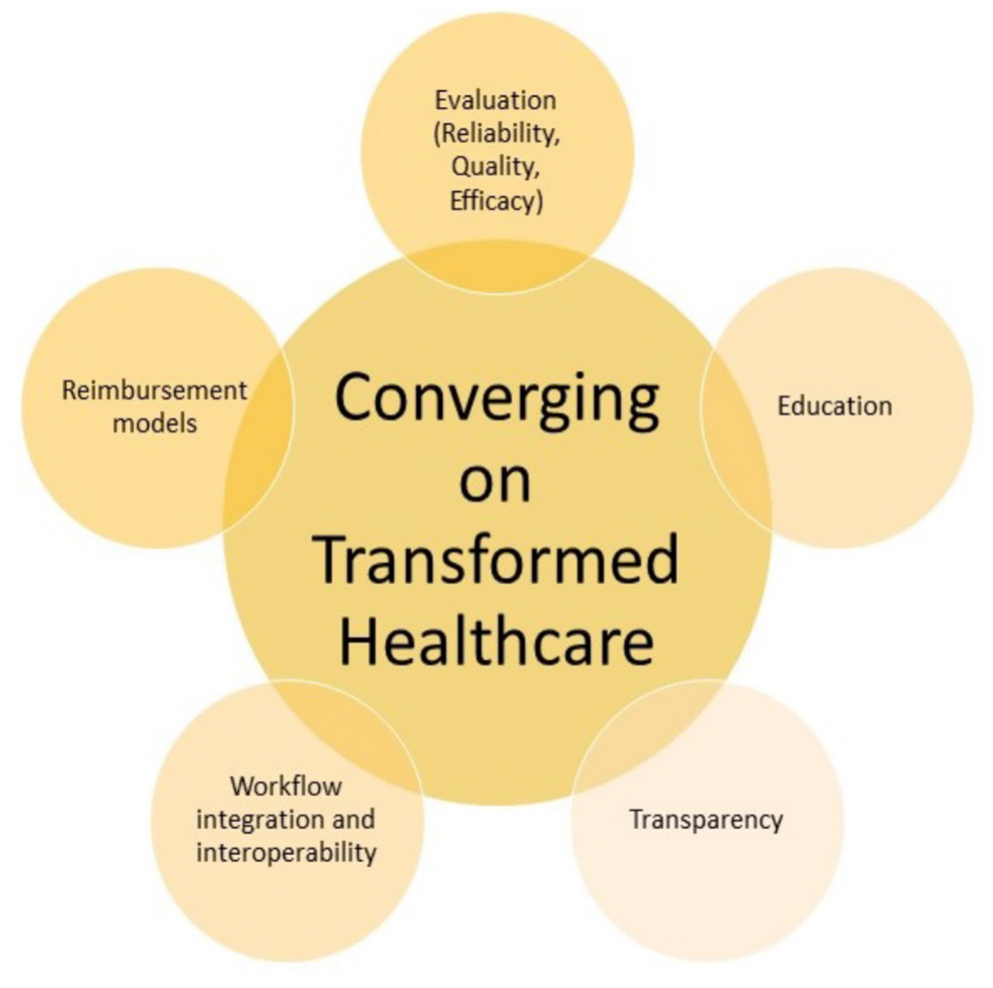
Roadmap for transformed health care.

Based on this analysis, what is the roadmap to success for these technologies, and how will they succeed in contributing to the future of health care? Figure 5 depicts the convergent approaches that need to be developed to ensure safe and productive adoption, in line with the P5 medicine principles.

First, AI technology is currently undergoing a remarkable revival and being applied to many domains. Health applications will both benefit from and contribute to further advances. In areas such as image classification or natural language understanding, both of which have obvious utility in health care, the rate of progress is remarkable. Today's AI techniques may seem obsolete in ten years.

Second, the more technical challenges of AI–such as privacy, explainability, or fairness–are being worked on, both in the research community and in the legislative and regulatory world. Standard procedures for assessing the efficacy and safety of systems will be needed, but in reality, this is not a new concept: it is what has been developed over the years to approve new medicines. We need to be consistent and apply the same hard-headed validation processes to the new technologies.

Third, it should be clear from our exploration of this subject that *education*–of patients as well as of professionals–is key to the societal acceptance of the role that AI and robotics will be called upon to play. Every invention or innovation–from the steam engine to the telephone to the computer–has gone through this process. Practitioners must learn enough about how AI models and robotics work to build a “working relationship” with those tools and build trust in them–just as their predecessors learned to trust what they saw on an X-ray or CT scan. Patients, for their part, need to understand what AI and robotics can or cannot do, how the physician will remain in the loop when appropriate, and what data is being collected about them in the process. We will have a responsibility to ensure that complex systems that patients do not sufficiently understand cannot be misused against them, whether accidentally or deliberately.

Fourth, health care is also a business, involving financial transactions between patients, providers, and insurers (public or private, depending on the country). New cost and reimbursement models will need to be developed, especially given that when AI is used to assist professionals, not replace them, the cost of the system is additive to the human cost of assessing the data and reviewing the system's recommendations.

Fifth and last, clinical pathways have to be adapted and new role models for physicians have to be built. Clinical paths can already differ and make it harder to provide continuity of care to a patient who moves across care delivery systems that have different capabilities. This issue is being addressed by the BPM+ Health Community^14^ using the business process, case management and decision modeling standards of the Object Management Group (OMG). The issue will become more complex by integrating AI and robotics: every doctor has similar training and a stethoscope, but not every doctor or hospital will have the same sensors, AI programs, or robots.

Eventually, the convergence of these approaches will help to build a complete digital patient model–a digital twin of each specific human being – generated out of all the data gathered from general practitioners, hospitals, laboratories, mHealth apps, and wearable sensors, along the entire life of the patient. At that point, AI will be able to support superior, fully personal and predictive medicine, while robotics will automate or support many aspects of treatment and care.

## Data Availability Statement

The original contributions presented in the study are included in the article/supplementary material, further inquiries can be directed to the corresponding author.

## Author Contributions

KD came up with the classification of AI and robotic systems. CB identified concrete application examples. Both authors contributed equally, identified adoption challenges, and developed recommendations for future work. Both authors contributed to the article and approved the submitted version.

## Conflict of Interest

The authors declare that the research was conducted in the absence of any commercial or financial relationships that could be construed as a potential conflict of interest.

## Publisher's Note

All claims expressed in this article are solely those of the authors and do not necessarily represent those of their affiliated organizations, or those of the publisher, the editors and the reviewers. Any product that may be evaluated in this article, or claim that may be made by its manufacturer, is not guaranteed or endorsed by the publisher.

## Footnotes

^1^ https://cmte.ieee.org/futuredirections/2019/07/21/autonomous-systems-in-healthcare/

^2^ https://emag.medicalexpo.com/ai-powered-chatbots-to-help-against-self-isolation-during-covid-19/

^3^ https://www.med.ubc.ca/news/sepsis-leading-cause-of-death-worldwide/

^4^ https://www.sepsis.org/wp-content/uploads/2017/05/Sepsis-Fact-Sheet-2018.pdf

^5^ https://medcitynews.com/2021/07/johns-hopkins-spinoff-looking-to-build-better-risk-prediction-tooing,ls-emerges-with-15m/

^6^ https://www.patchdmedical.com/

^7^ https://hisigma.mit.edu

^8^ https://blog.radiology.virginia.edu/covid-19-and-imaging/

^9^ https://hitinfrastructure.com/news/diagnostic-robotics-mayo-clinic-bring-triage-platform-to-patients

^10^ https://www.exer.ai

^11^ https://www.gcmas.org/map

^12^ https://www.media.mit.edu/groups/biomechatronics/overview/

^13^ https://digital-strategy.ec.europa.eu/en/policies/regulatory-framework-ai

^14^ https://www.bpm-plus.org/
